# Inhibitor of cyclooxygenase-2 induces cell-cycle arrest in the epithelial cancer cell line via up-regulation of cyclin dependent kinase inhibitor p21

**DOI:** 10.1038/sj.bjc.6600183

**Published:** 2002-04-08

**Authors:** T Toyoshima, R Kamijo, K Takizawa, K Sumitani, D Ito, M Nagumo

**Affiliations:** Second Department of Oral and Maxillofacial Surgery, School of Dentistry, Showa University, 2-1-1, Kitasenzoku, Ota-ku, Tokyo 145-8515, Japan; Department of Biochemistry, School of Dentistry, Showa University, 1-5-8, Hatanodai, Shinagawa-ku, Tokyo 142-8555, Japan

**Keywords:** cyclooxygenase-2, squamous cell carcinoma, NS-398, G0/G1 arrest, p21

## Abstract

Cyclooxygenase-2 is the rate-limiting enzyme in synthesis of prostaglandins and other eicosanoids. Prior reports have shown that inhibition of cyclooxygenase-2 activity, either by selective inhibitors or by antisense oligonucleotide, results in suppression of growth of squamous cell carcinoma cell lines which express high cyclooxygenase-2 levels, such as NA, a cell line established from a squamous cell carcinoma of the tongue. To investigate the mechanisms by which cyclooxygenase-2 inhibitors suppressed growth of these cells, the effects of NS-398, the selective cyclooxygenase-2 inhibitor, on cell-cycle distribution were examined. NS-398 induced G0/G1 cell-cycle arrest in NA cells which expressed cyclooxygenase-2. G0/G1 arrest induced by NS-398 was accompanied by up-regulation of cyclin-dependent kinase inhibitor p21, but not by up-regulation of the other cyclin-dependent kinase inhibitors. Transfection with p21 antisense oligonucleotide inhibited cell-cycle arrest induced by NS-398. Accumulation in G0/G1 was also observed in NA cells transfected with cyclooxygenase-2 antisense oligonucleotide. On the other hand, NS-398-treated NA cells showed a loss of plasma membrane asymmetry, a marker of early events in apoptosis. However, NS-398 did not induce other morphological and biochemical changes related to apoptotic cell death. These results suggest that cyclooxygenase-2 inhibitor induces G0/G1 cell-cycle arrest in NA cells by up-regulation of p21. Our results also suggest that NS-398 is not sufficient to complete the whole process of apoptosis in NA cells, although it induces an early event in apoptosis.

*British Journal of Cancer* (2002) **86**, 1150–1156. DOI: 10.1038/sj/bjc/6600183 www.bjcancer.com

© 2002 Cancer Research UK

## 

Prostaglandin endoperoxide H synthase (also referred to as cyclooxygenase (COX)) is the rate-limiting enzyme in the production of prostaglandins (PG) and thromboxanes, which are involved in various physiologic and pathologic processes such as inflammation, angiogenesis and development of cancer. Two COX isoforms (COX-1 and COX-2) that differ significantly in their patterns of expression have been identified (Kujubu *et al*, 1991; Xie *et al*, 1991; Hla and Neilson, 1992; O'Banion *et al*, 1991; Williams and Shacter, 1997). COX-1 is constitutively expressed in many tissues and cells, including the gastric mucosa (Smith *et al*, 1993; Williams and DuBois, 1996). By contrast, COX-2 is an immediate, early response gene expressed at very low levels unless induced by mitogens (Lee *et al*, 1992), cytokines (Maier *et al*, 1990), and tumour promoters (Kujubu *et al*, 1991). After these stimulations, cells expressing COX-2 synthesise and release large amount of prostanoids. These COX isoforms have both overlapping as well as distinct physiologic and pathologic functions (Hia *et al*, 1993; Dubois *et al*, 1998). While COX-1 is involved in the homeostasis of various physiologic functions, COX-2 is responsible for many inflammatory processes. Both COX enzymes catalyse two distinct reactions: (1) conversion of arachidonic acid to PGG_2_ via the cyclooxygenase activity and (2) the reduction of PGG_2_ to PGH_2_ via the peroxidase activity. PGH_2_ is converted by distinct isomerases into biologically active PGs, including PGD_2_ and PGE_2_. Nonsteroidal anti-inflammatory drugs such as aspirin and indomethacin inhibit the cyclooxygenase activity, but not the peroxide activity (Lahenville *et al*, 1994; Smith and Dewitt, 1996). Recently, inhibitors which selectively inhibit COX-2 activity have been made (Taketo, 1998a,b). These inhibitors, for example NS-398, inhibit the cyclooxygenase activity of COX-2 and thus inhibit prostanoid synthesis (Warner *et al*, 1999).

It is reported that greater than 80% of colon cancers in humans have increased COX-2 levels when compared to adjacent normal tissue (Williams *et al*, 1997), and overexpression of COX-2 has been identified as an early central event in colon carcinogenesis (Kargman *et al*, 1995; Tsuji and Dubois, 1995; Boolbol *et al*, 1996; Reddy *et al*, 1996; Tsuji *et al*, 1997). Previous studies have also demonstrated that constitutive expression of COX-2 in human colon cancer cells promotes tumour invasion and the metastatic potential of these cells, and have suggested that COX-2 selective inhibitors can be suitable chemopreventive agents for colorectal cancer (Oshima *et al*, 1996; Tsuji *et al*, 1997).

As well as colorectal cancer, increased COX-2 expression is found in carcinomas of various organs including the breast, prostate (Gupta *et al*, 2000), lung (Wolff *et al*, 1998), oesophagus (Zimmermann *et al*, 1999), pancreas (Tucker *et al*, 1999) and mucous membrane of head and neck (Chan *et al*, 1999). Thus it is suggested that selective COX-2 inhibitors may be effective chemopreventive agents for these carcinomas.

Recently, we and others have shown that COX-2 selective inhibitor (NS-398) inhibits proliferation of head and neck squamous cell carcinoma (SCC) cell lines expressing COX-2 mRNA (Higashi *et al*, 2000; Sumitani *et al*, 2001). Suppression of proliferation was also observed in these cells transfected with COX-2 antisense oligonucleotide. However, the relevant mechanisms by which COX-2 inhibition resulted in inhibition of proliferation of these cells has not been well defined. The growth inhibition in cancer cells is, in principle, associated withdrawal from the cell cycle. The cell cycle is regulated by proteins known as cyclins and their associated cyclin-dependent kinases (CDKs). Mammalian cells contain at least 11 cyclins and five CDKs, and specific cyclin/CDK complexes regulate the different cell cycle checkpoints (Nurse 1994). One of the most important checkpoints occurs in the late G1, just before the start of DNA replication. D-type cyclins associate with and activate CDK2 and CDK4 proteins, which allow cells to pass this restriction point. Loss of this cell-cycle checkpoint has been linked to cancer, in many cases through changes in the cyclin/CDK complexes (Hunter and Pines, 1994). More recently, a new class of cell-cycle regulator, CDK inhibitors (CKIs), has been identified. CKIs bind to and inhibit the activity of cyclin/CDK complexes, resulting in the inhibition of cell-cycle progression. Two structurally defined classes of CKIs have been identified. The first class, termed the p21 family, includes p21^*WAF1*^, p27^*KIP1*^ and p57^*KIP2*^, which inhibit the activity of G1- and S phase and to a lesser extent the mitotic cyclin/CDK complexes. The second class of CKIs, termed the INK4 family, includes p16^*INK4A*^, p15^*INK4B*^, p18^*INK4C*^ and p19^*INK4D*^, which inhibit G1-specific cyclin D-CDK4/6 kinase activity only.

It was reported that COX-2 inhibitor suppressed proliferation of these cells via reduction of prostanoid production which affected cell proliferation, tumour growth and immune responsiveness (Hia *et al*, 1993). However, COX isoforms possess a separate peroxidase activity that can modulate other cellular signalling pathways such as NF-κB (Munroe *et al*, 1995). It has been shown that overexpression of COX-1 resulted in the tomorigenic transformation of ECV-304 cells, and that it was not inhibited by Indomethacin (Narko *et al*, 1997). Simmons and colleagues showed that the COX-2 protein bound to an apoptosis and autoimmunity-associated protein termed nucleobindin (Ballif *et al*, 1996). These results raise the possibility that COX-2 may regulate intercellular signalling by both PG-dependent and PG-independent actions. In this study we examined the effects of inhibition of COX-2, either by selective inhibitor (NS-398) or transfection of COX-2 antisense oligonucleotide, on the cell cycle distribution of NA, an SCC cell line established from the tongue. The effect of NS-398 on induction of apoptosis in NA cells was also investigated.

## MATERIALS AND METHODS

### Reagents and Antibodies

NS-398, a selective inhibitor of COX-2, was purchased from Calbiochem (La Jolla, CA, USA). Nitric oxide (NO) spontaneous donor, NOC-12, was obtained from Dojindo Laboratories (Kumamoto, Japan). Triton X-100 (polyoxyethylene (10) octylprenyl ethel) was purchased from Wako Pure Chemical Industries, Ltd. (Osaka, Japan) Phenylmethylsulphonyl fluoride (PMSF), leupeptin and approtinin were purchased from Sigma (St Louis, Missouri, USA). Unconjugated polyclonal (p) antibodies (Ab) against the following human antigens were used in this study: Anti-p21 pAb (rabbit (r) immunoglobulin (Ig) G, C-19; Santa Cruz Biotechnology, Santa Cruz, CA, USA) and p27 pAb (rIgG, N-20; Santa Cruz Biotechnology, Santa Cruz, CA, USA).

### Cell line and cell culture

NA, a cancer cell line established from a patient with SCC of the tongue, was maintained as monolayers in Dulbecco's modified Eagle's medium (DMEM) supplemented with 10% heat inactivated foetal bovine serum (FBS), 100 u ml^−1^ penicillin and 100 μg ml^−1^ streptomycin (complete medium). Subconfluent monolayers of NA cells were employed in all experiments.

### Cell-cycle analysis

NA cells were trypsinized and 10^6^ cells were plated. Eighteen hours after incubation, NS-398 was added to the culture, and cells were further incubated for 24 h. Cell cycle analysis was performed on these cells using DNA staining and flow cytometry. The cells were washed twice with PBS, treated with 0.2% of TritonX-100 and 0.5% of RNase, and stained with 50 μg ml^−1^ of propidium iodide (PI). The relative DNA content per cell was obtained by measuring the fluorescence of PI that bound stoichiometrically to DNA. The cell cycle was analysed by ModFit LT software (Verity Software, Inc.).

### Western blot analysis

NA cells were plated in 10 ml of complete medium containing 2×10^6^ cells. Eighteen hours after incubation, NS-398 was added to the culture. At 6 and 12 h after incubation, the cells were lysed with lysis buffer (10 mM Tris-HCl (PH 7.5), 150 mM NaCl, 5 mM EDTA, 1% TrItonX-100, 1 mM PMSF, 10 mg ml^−1^ Leupeptin, 20 mg ml^−1^ aprotinin) at 4°C. After freezing and thawing three times, insoluble material was removed by centrifugation at 15 000 **g** for 15 min at 0°C. Proteins from cell lysates (30 μg) were separated on acrylamid-bisacrylamide-sodium dodecyl sulphate gels in running buffer (25 mM Tris base, 192 mM glycine, 0.1% SDS) and electrophoretically transferred to Hybond-P membranes (Amersham Pharmacia Biotech, Buckinghamshire, UK) in transfer buffer (25 mM Tris base, 0.19 mM glycine, 10% methanol, 0.05% Triton X100). The membranes were blocked in blocking solution (20 mM Tris-HCl (pH 7.4), 0.15 M NaCl, 5% nonfat dry milk) for 1 h to overnight at room temperature. The blots were then incubated with first antibodies in antibody solution (20 mM Tris-HCl (pH 7.4), 0.15 M NaCl, 0.05% Triton X-100) with gentle agitation for 1 h to overnight at room temperature. After incubation with second antibody (1 : 2500) for 1 h at room temperature, the blots were determined using an ECL Western blotting kit (Amersham Pharmacia Biotech, Buckinghamshire, UK), according to the manufacturer's instructions.

### Phosphorothioate antisense oligonucleotide studies

NA cells were cultured in 10 ml of complete medium containing 5×10^5^ cells in the presence of p21 antisense oligonucleotides or sense oligonucleotides (10 μM). Twenty-four hours after incubation, NA cells were cultured in the presence of NS-398 (79.5 μM) at 37°C for 24 h, and cell cycle analysis was performed as described above.

NA cells were also incubated with COX-2 phosphorothioate antisense or sense oligonucleotide for 24 h. The cell cycle distribution of NA cells which were transfected with COX-2 antisense oligonucleotides (10 μM) was analysed by flow cytometry, and the results were compared to the untransfected and sense-transfected NA cells. The phosphorothioate oligonucleotides used in this study were as follows: COX-2 phosphorothioate antisense oligonucleotide (5′-CAGTTCAGTCGAACGTTCTTTTAGTAGTAC -3′), COX-2 phosphorothioate sense oligonucleotide (5′-GTACTACTAAAAGAACGTTCGACTGAACTG -3′), p21 phosphorothioate antisense oligonucleotide (5′-TCCCCAGCCGGTTCTGACAT-3′), p21 phosphorothioate sense oligonucleotide (5′-ATGTCAGAACCGGCTGGGGA-3′) (Hokkaido System Science, Sapporo, Japan).

### Detection of apoptosis by annexin V staining and nuclear staining

NA cells were incubated in the presence or absence of NS-398. Forty-eight hours after incubation, the annexin V-affinity assay and nuclear staining were performed on NA cells as described before. Briefly, NA cells were washed with binding buffer and incubated with FITC-labelled annexin V in binding buffer (TACS Annexin V-FITC kit, Trevigen, Gaitherburg, USA) for 20 min at 20°C in the dark. Cells were washed three times, harvested by gentle scraping, and resuspended in PBS with 1.5 mM Ca^2+^ and 1% FBS. Cells were then fixed with 4% paraformaldehyde to prevent aggregation, and were analysed by flow cytometry (FACS Calibur, Becton-Dickinson). Data were analysed using the Cell Quest 3.1 software. The percentage of annexin V-positive cells was determined after setting appropriate markers for negative and positive populations.

For assessment of the appearance of typical morphological changes of apoptosis, staining of the cells with the DNA-specific fluorochrome bis-benzimide trihydrochloride (H33258, Calbiochem, La Jolla, CA, USA) was performed on NA cells. After treatment with NS-398 for 48 h, NA cells were fixed in PBS containing 0.25% glutaraldehyde at pH 7.2, stained with 2 mg ml^−1^ of bis-benzimide, and analysed under an inverted fluorescence microscope. Cells with condensed and fragmented nuclei were considered apoptotic.

## RESULTS

### Cell-cycle analysis of NA cells treated with NS-398

We recently reported that COX-2 expression was enhanced in NA cells, and that NS-398 suppressed proliferation of NA cells via a PG-dependent pathway (Sumitani *et al*, 2001). The growth inhibitory effect of NS-398 was dose-dependent. The suppression of proliferation by NS-398 was time-dependent. It was observed to be slight on days 1 and 2, and become obvious on days 3 and 4. The viability of NA cells exposed to NS-398 for 4 days was approximately 99%, as determined by trypan blue dye exclusion assay (Sumitani *et al*, 2001).

**Figure 1:**
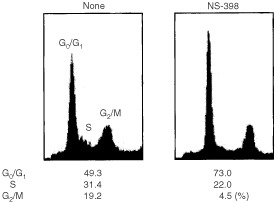
Effect of NS-398 on the cell cycle distribution in NA cells. NA cells were incubated in the presence or absence of NS-398 (79.5 μM) for 24 h. Cell cycle analysis was performed as described in Materials and Methods. Results from a representative analysis are shown.

To determine whether suppression of proliferation of NA cells induced by NS-398 is associated with cell-cycle arrest, NA cells were incubated in the presence of NS-398 for 24 h. According to the manufacturer's information, NS-398 at 79.5 μM selectively inhibits COX-2 activity without inhibiting COX-1 activity. Therefore NS-398 at 79.5 μM was employed in this study. The cell-cycle distribution was then analysed by DNA staining with PI followed by flow cytometrical analysis. Treatment with NS-398 induced a decrease in S phase population (from 31.4 to 22.0%) and a significant increase in G0/G1 population (49.3% to 73.0%), compared with untreated NA cells (Figure 1

).

### Effect of NS-398 on the expression of CKIs in NA cells

**Figure 2:**
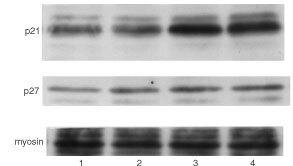
Western blot analysis of p21 expression in NA cells. NA cells were incubated in the presence (lanes 3, 4) or absence (lanes 1, 2) of NS-398 (79.5 μM). At 6 (lanes 1, 3) and 12 h (lanes 2, 4), NA cells were lysed, and Western blot analysis was performed as described in Materials and Methods.

The cell-cycle progression of many types of cells is negatively regulated by the series of proteins called CKIs. Therefore the effect of NS-398 on the expression of CKIs in NA cells was investigated. NA cells were incubated in the presence or absence of NS-398. At 6 and 12 h after incubation, cells were lysed and CKI protein levels were determined by Western blot analysis. p21 was slightly expressed in untreated NA cells, and was up-regulated by NS-398. The up-regulation of p21 became obvious at 6 and 12 h after incubation with NS-398 (Figure 2

), and was seen in longer incubation periods being tested (data not shown). Densitometrical analysis revealed that p21 level in cells treated with NS-398 was higher than that in untreated cells (3.2-fold higher at 6 h treatment, and 1.7-fold higher at 12 h treatment) (data not shown). The other p21 family of CKI, p27, was also detected in untreated NA cells, but it was not up-regulated by NS-398 (Figure 2). p57 was slightly expressed in untreated NA cells. As well as p27, p57 was not up-regulated by NS-398 (data not shown). We next examined the expression of the INK4 family of CKI in NA cells in the presence or absence of NS-398. In contrast to the p21 family of CKI, none of them were detected in untreated NA cells, and they were not induced by NS-398 (date not shown).

### Cell-cycle analysis of NA cells after transfection of p21 phosphorothioate antisense oligonucleotides

To confirm that the cell cycle arrest induced by NS-398 was due to up-regulation of p21, we employed studies with p21 phosphorothioate antisense oligonucleotide. NA cells were pretreated with p21 phosphorothioate antisense oligonucleotide (10 μM). Twenty-four hours after incubation, cells were further incubated with or without NS-398 for 24 h. The cell cycle distribution of these cells was analysed by flow cytometry, and the results were compared to the cell cycle distribution of untransfected, p21 sense and scrambled oligonucleotide transfected cells and untreated cells. Transfection of p21 antisense oligonucleotide could induce complete inhibition of the G0/G1 cell cycle arrest induced by NS-398 (Table 1

**Table 1:**
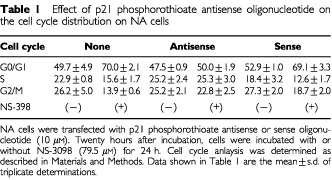
Effect of p21 phosphorothioate antisense oligonucleotide on the cell cycle distribution on NA cells

). Both sense (Table 1) and scrambled (data not shown) oligonucleotides did not induce inhibition of the G0/G1 cell cycle arrest induced by NS-398. Western blot analysis showed that treatment with p21 antisense oligonucleotides suppressed p21 protein levels in NA cells (3.34-fold reduction as determined by densitometry), confirming that p21 antisense oligonucleotide employed here is working in NA cells.

### Cell-cycle analysis of NA cells after transfection of COX-2 phosphorothioate antisense oligonucleotides

**Figure 3:**
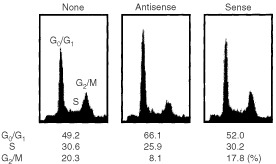
Effect of COX-2 phosphorothioate antisense oligonucleotide on the cell cycle distribution of NA cells. NA cells were transfected with COX-2 phosphorothioate antisense or sense oligonucleotide (10 μM) for 24 h. Cell cycle analysis was performed as described in Materials and Methods. Results from a representative analysis are shown.

To further assess whether G0/G1 cell-cycle arrest induced by NS-398 is associated with inhibition of COX-2 expression, NA cells were transfected with COX-2 phosphorothioate antisense oligonucleotides and the cell-cycle distribution was compared to the cell-cycles of untransfected and COX-2 sense oligonucleotide-transfected NA cells. Although the control COX-2 sense oligonucleotide had no effect on the cell-cycle distribution of NA cells, transfection with COX-2 antisense oligonucleotides into NA cells was associated a with decrease in S phase population (30.6 to 25.9%) and a increase in GO/G1 population (49.2 to 66.1%) compared with untransfected NA cells (Figure 3

). Western blot analysis showed that p21 level in COX-2 antisense-treated cells was higher than that in sense-treated cells (data not shown). The effect of COX-2 antisense oligonucleotides on p21 up-regulation was weaker than that of NS-398. Western blot analysis revealed that treatment with COX-2 antisense oligonucleotide up-regulated p21 level in NA cells (2.4-fold up-regulation as determined by densitometry). These result indicates that p21 is really one of the downstream elements for COX-2 associated growth suppression.

### Apoptosis assay

We next examined whether the treatment with NS-398 induced apoptotic cell death in NA cells, using two independent techniques: annexin V affinity assay, and staining with a DNA-specific dye.

It is reported that plasma membrane changes occur very early in the cell undergoing apoptotic cell death. Recent studies indicate that apoptotic cell death is accompanied by surface exposure of phosphatydylserine (PS) which locates on the inner surface of the plasma membrane, while the membrane integrity remains unchanged. Surface exposed PS can be detected by its affinity for annexin V, a phospholipid binding protein, and it is therefore shown to be a useful marker of early apoptoic change (Von England *et al*, 1998).

NA cells were incubated with NS 398 for 24, 48 and 72 h, and cells were labelled with annexin V-FITC and PI. Flow cytometry was performed on a FACS Calibur, and data were analysed by the Cell Quest 3.1 software. The per cent of Annexin V positive cells was dramatically increased by NS-398 for 24 h (from 3.9 to 28.1%) (Table 2

**Table 2:**
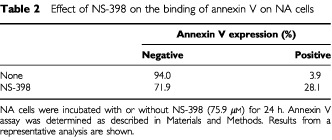
Effect of NS-398 on the binding of annexin V on NA cells

). The per cent of Annexine V positive cells was increased compared to control, but gradually decreased after 24 h (data not shown).

**Figure 4:**
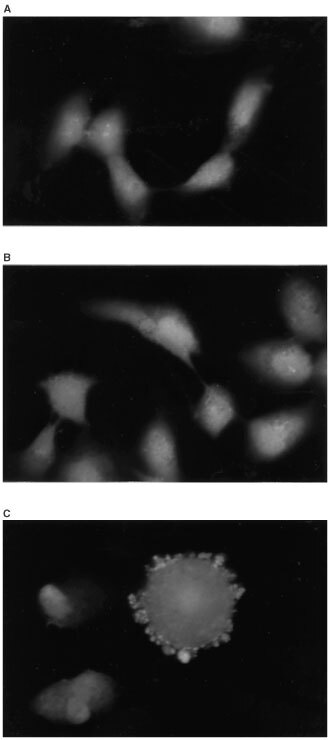
Nuclei of NS-398-treated NA cells. NA cells were incubated in the presence (**B**) or absence (**A**) of NS-398 for 24 h. Apoptoic cell death was detected as described in Materials and Methods. NA cells treated with apoptosis inducing agent (NOC12, nitric oxide donor) for 24 h are shown as a positive control (**C**).

However, treatment of NA cells with NS-398 for 24, 48 and 72 h did not result in the appearance of typical morphological changes of apoptosis on staining the cells with the DNA-specific fluorochrome bis-benzimide trihydrochloride, as demonstrated in Figure 4

.

## DISCUSSION

An expanding body of information has suggested the possible application of COX-2 selective inhibitors in cancer chemoprevention. Recent studies revealed that COX-2 was overexpressed in SCC cells, and that a selective COX-2 inhibitor suppressed proliferation of SCC cell lines. This inhibition was mediated by reduced synthesis of PGE_2_, which plays important roles in the proliferation of cells. Here we showed that growth inhibition of NA cells by NS-398 was associated with G0/G1 cell-cycle arrest. Western blot analysis showed that NS-398 up-regulated p21 protein, a specific inhibitor of CDKs, in NA cells. Moreover, growth inhibition induced by NS-398 was reduced in p21-antisense treated NA cells compared to untreated NA cells. Taken together, the accumulation in G0/G1 by NS-398 might be mediated by up-regulation of p21.

The effect of NS-398 was specific for p21, since the expression of other CKIs was not affected by NS-398. The expression of D-type cyclins (cyclin D1, D2, D3) and their associating kinases (CDK2 and 4), which allow cells to pass the G1 checkpoint, were also not altered by NS-398 in NA cells (data not shown). Recent study has demonstrated that NS-398 induced G1 growth arrest in A549 lung cancer cells. NS-398 specifically up-regulated cyclin-dependent kinase inhibitor p27, whereas the expression of G1 cyclins and CDKs were not changed (Hung *et al*, 2000). These results were not consistent with our results. Further investigation should be necessary to elucidate these inconsistencies.

The mechanism by which NS-398 up-regulates p21 protein remains to be examined. The p21 protein was identified originally as a gene that can be directly regulated by the tumor suppressor protein p53 (El-Deiry *et al*, 1994; Hunter and Pines, 1994; Sherr and Roberts, 1995; Harper and Elledge, 1996). The expression of p21 is stimulated by a variety of external stimuli, such as various growth factors, cytokines, tumor promoters and DNA damaging agents (El-Deiry *et al*, 1994; Sheikh *et al*, 1994). However not all stimulatory pathways involve the p53 protein, as some of the agents are also able to induce p21 expression in p53-negative cells (Michieli *et al*, 1994; El-Deiry *et al*, 1995; Parker *et al*, 1995; Alpan and Pardee 1996; Li *et al*, 1995; Liu *et al*, 1996; Zeng and El-Deiry, 1996; Prowse *et al*, 1997). DNA sequence analysis of exons 2 to 11 of the p53 gene revealed one missense mutation in exon 6 (codon 220, Tyr to Hys) of NA cells (data not shown). Codon 220 is located at the DNA binding domain of p53 gene, and this mutation appears to inhibit specific DNA binding (Gottlieb and Oren, 1996). Accordingly, the up-regulation of p21 in NS-398-treated NA cells might be mediated by a p53-independent pathway.

Evidence suggests that the increase in tumorigenic potential by COX-2 overexpression is associated with resistance to apoptosis (Tsuji and Dubois, 1995). Selective inhibitors of COX-2 have been demonstrated to induce apoptosis in a variety of cancer cells, including those of the colon, stomach, prostate and breast. These observations are consistent with the COX-2 inhibitor being a chemopreventive agent that increases the susceptibility of cancer cells to apoptosis. However, NS-398 could only induce a very early stage of apoptosis (detection of annexin V) in NA cells, and it could not induce morphological change (nuclear fragmentation) which was related to apoptotic cell death. To further investigate the effect of NS-398 on apoptotic process in NA cells, NA cells were incubated with or without NS-398 for 6, 12 and 24 h, and caspase activities (caspases 1, 3 6, 8 and 9) were measured using specific ELISA (Medical and Biological Laboratories, Tokyo, Japan). No caspase activities were induced by NS-398 (data not shown). These results suggest that COX-2 inhibitor is not sufficient to complete the whole process of apoptosis in SCC cells. The reason why COX-2 inhibitor cannot induce apoptotic cell death in NA cells remains elusive. Considering the molecular basis for COX-2 inhibitor-induced apoptosis, it has been proposed that it might be due to the down-regulation of Bcl-2 expression (Liu *et al*, 1998; Sheng *et al*, 1998). It has also reported that apoptotic effect of celecoxib, a selective COX-2 inhibitor, is partly mediated by blocking the activation of the anti-apoptotic kinase AKT (Hsu *et al*, 2000). However, neither down regulation of Bcl-2 or blocking activation of AKT was observed in NS-398 treated NA cells (data not shown).

It is generally accepted that COX-2 inhibitors exert their action via blocking PG synthesis by direct COX inhibition. However, whether COX-2 inhibitors block proliferation of cancer cells solely by blocking PG synthesis is a matter still being discussed. Several studies have shown that COX-2 inhibitors can act through mechanisms that are independent of their ability to inhibit COX-2. For example, celecoxib, a selective COX-2 inhibitor, induced apoptosis of a prostate cancer cell line through a target other than COX-2 (Hsu *et al*, 2000). COX-2 inhibited cell cycle progression in a variety of cell types by a novel mechanism that did not require the synthesis of PG (Trifan *et al*, 1999). Recently we have shown that NS-398 inhibits the proliferation and differentiation of human leukemia cell lines via PG-independent pathways (Nakanishi *et al*, 2001). Therefore, it is indicated that selective COX-2 inhibitors also possess COX-2 independent pathways which are responsible for their functions. When NA cells were treated with COX-2 antisense oligonucleotide, the cell cycle distribution of NA cells was accumulated to G0/G1. Moreover, the degree of suppression of proliferation with NS-398 was almost the same as that with COX-2 antisense oligonucleotide. This result indicates that accumulation in G0/G1 by NS-398 is via decreased PG biosynthesis resulting from down-regulation of COX-2 expression, since PGE2 induced proliferation of NA cells (Sumitani *et al*, 2001). Further investigations are required to identify, at a molecular level, the mechanism of the cell-cycle arrest by COX-2 inhibitor, and to assess its clinical relevance.
